# Separate Kingdoms, Same Conspiracies: Conserved Viral Strategies for Immune Evasion in Animal and Bacterial Hosts

**DOI:** 10.1002/mco2.70215

**Published:** 2025-05-10

**Authors:** Junyi Wang, Xiang He, Guoping Li

**Affiliations:** ^1^ Laboratory of Allergy and Precision Medicine, Department of Respiratory Medicine Chengdu Institute of Respiratory Health, Affiliated Hospital of Southwest Jiaotong University, the Third People's Hospital of Chengdu Chengdu China

1

In a recent study published in *Cell*, Hobbs et al. uncovered a strikingly conserved immune evasion strategy shared by animal poxviruses and bacteriophages, involving the degradation of host cyclic nucleotide signals through cyclic GMP–AMP (cGAMP) phosphodiesterases (PDEs) [1]. This discovery highlights the evolutionary convergence across kingdoms, providing new insights into viral pathogenesis and opening avenues for developing broad‐spectrum antiviral therapeutics that target these conserved pathways.

Animal and bacterial cells have evolved complex antiviral defense systems to sense viral invaders and initiate immune responses. A key component of these defense systems is the cyclic GMP–AMP synthase (cGAS)—stimulator of interferon genes (STING) pathway in animals, which is responsible for detecting viral DNA and generating second messengers like 2′3′‐cGAMP, leading to the production of interferons and other antiviral molecules [2]. In bacteria, a similar pathway exists, known as cyclic oligonucleotide‐based anti‐phage signaling systems (CBASS), which use cyclic nucleotides, such as 3′3′‐cGAMP, to initiate antiviral responses, including cell death to prevent phage propagation [3]. However, recent studies have further demonstrated that diverse strategies employed by phages to inhibit or evade CBASS‐mediated immunity, underscoring the ongoing evolutionary arms race between hosts and viruses [4].

Hobbs et al. focused on two classes of viral proteins: cGAMP PDEs from animal poxviruses and the Anti‐CBASS1 (Acb1) proteins from bacteriophages [1]. Acb1 is a phage immune evasion protein that specifically degrades bacterial cyclic nucleotide immune signals, thereby inhibiting host CBASS systems [5]. Interestingly, poxvirus cGAMP PDE and phage Acb1 share a striking structural and functional resemblance [1]. The viral proteins share a highly conserved phosphoesterase fold and aligned active‐site residues, reflecting architectural and catalytic homology between them. In addition, both poxvirus cGAMP PDE and phage Acb1 proteins use a conserved set of water molecules to coordinate the cyclic nucleotide substrates, enabling efficient cleavage of the phosphodiester bond. Moreover, both viral proteins feature a lid domain—located at the N‐terminus in poxvirus PDEs and the C‐terminus in bacteriophage Acb1—that stabilizes the substrate within the active site to enhance catalytic efficiency [1]. Strikingly, recombinant bacteriophages engineered by Hobbs et al., carrying the poxvirus PDE gene instead of their native *acb1* gene, successfully evaded bacterial CBASS defenses [1]. The fact that a eukaryotic viral protein can function in a prokaryotic system suggests that these immune evasion mechanisms are not limited by the host species. Instead, they exploit conserved biochemical pathways that are fundamental to antiviral defense across biological kingdoms.

The biological implications of this research are profound. The discovery that poxvirus cGAMP PDE and bacteriophage Acb1 can both degrade cyclic nucleotide signals suggests that these enzymes are highly adaptable, capable of targeting a wide range of nucleotide immune signals. This adaptability may give these viruses a significant advantage in evading host immune responses, allowing them to infect a broader range of hosts. Moreover, the study's cross‐kingdom comparisons reveal that while both viral types employ similar strategies, they have evolved distinct structural features that optimize their immune evasion in specific environments. This structural variation may reflect the different immune pressures faced by animal viruses compared to bacteriophages, which need to navigate the more complex immune systems of multicellular organisms. Additionally, the research highlights the potential for cross‐kingdom viral protein functionality. This cross‐kingdom activity opens up new avenues for research into how viral enzymes can be repurposed for use in different biological contexts, potentially leading to novel antiviral therapies that exploit these conserved immune evasion mechanisms.

Understanding the conserved nature of viral immune evasion mechanisms has significant clinical and therapeutic implications. On one hand, given the structural and functional similarities between poxvirus cGAMP PDE and bacteriophage Acb1, it is conceivable that inhibitors developed to target one of these enzymes could be effective against a wide range of viruses. This opens up the possibility of developing broad‐spectrum antiviral drugs that target cGAMP PDEs across different viral families. On the other hand, the study suggests that these viral enzymes could be engineered for therapeutic purposes. For example, recombinant versions of cGAMP PDEs could potentially be used to modulate immune responses in conditions where the immune system is overactive, such as autoimmune diseases. By selectively degrading immune‐signaling molecules like cGAMP, these enzymes could help to dampen excessive immune activation, providing a new strategy for controlling inflammation.

While the study by Hobbs et al. provides critical insights into the structural and functional conservation of viral immune evasion proteins, it also raises several important questions. One of the key challenges moving forward is to determine how widespread these mechanisms are among other viral families. Are there additional classes of viruses that use similar strategies to evade host immunity? Answering this question will require a broader survey of viral genomes and structures, coupled with functional studies to assess the prevalence of these mechanisms. Another challenge is the theoretical risk that inhibitors targeting viral cGAMP PDEs might inadvertently affect human PDEs in similar pathways, potentially disrupting crucial cellular functions. This aspect underscores the need for precise molecular tools that can differentiate between viral and host PDEs to avoid detrimental impacts on the host's immune system. Additionally, the ability of viruses to rapidly mutate and adapt could lead to the emergence of resistance against drugs targeting these PDEs, potentially rendering them ineffective over time. This highlights the need for ongoing surveillance of viral evolution and adaptation in response to therapeutic pressures. Finally, there is a need for in vivo studies to validate these findings in the context of viral infection. While the structural and biochemical analyses presented in this study are compelling, it remains to be seen how these proteins function during actual infections in animal or bacterial hosts. Understanding the dynamics of immune evasion in vivo will be critical for translating these findings into therapeutic applications.

Collectively, Hobbs et al.’s research offers a groundbreaking perspective on the conserved mechanisms of immune evasion employed by viruses across the eukaryotic and prokaryotic kingdoms (Figure 1). By elucidating the structural and functional similarities between poxvirus cGAMP PDE and bacteriophage Acb1, this study not only advances our understanding of viral pathogenesis but also opens up new possibilities for therapeutic intervention. The conservation of these mechanisms across biological kingdoms highlights the universality of viral strategies for survival, underscoring the importance of continued research into viral immune evasion and its implications for human health.

**FIGURE 1:**
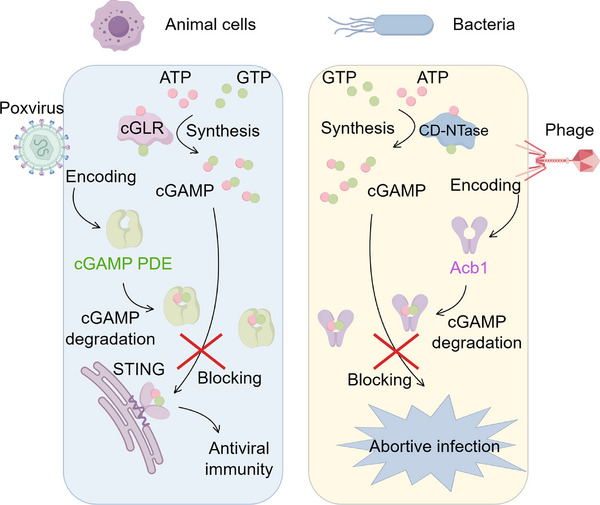
Poxviruses and bacteriophages use a conserved mechanism of immune evasion. In eukaryotic cells, the cGAS enzyme synthesizes cGAMP from ATP and GTP upon viral DNA detection, activating STING and leading to antiviral immunity. The poxvirus produces cGAMP PDE which degrades cGAMP, thereby counteracting this pathway. In bacteria, the CD‐NTase enzyme produces cGAMP, which activates defensive responses against bacteriophage infections. Phages evade this by encoding Acb1, a protein structurally similar to cGAMP PDE, which degrades cGAMP to prevent the abortive infection response. This figure was generated by Figdraw (www.figdraw.com). Acb1, anti‐CBASS1; ATP, adenosine triphosphate; CD‐NTase, cGAS/DncV‐like nucleotidyltransferases; cGAMP, 2′3′ cyclic GMP–AMP; cGAS, cyclic GMP–AMP synthase; cGLR, cGAS‐like receptor; GTP, guanosine triphosphate; PDE, phosphodiesterase; STING, stimulator of interferon genes.

## Author Contributions

Junyi Wang, Xiang He, and Guoping Li wrote and revised the manuscript. Junyi Wang drew the figure and made the artwork with input from all co‐authors. All the authors have read and approved the article.

## Ethics Statement

The authors have nothing to report.

## Conflicts of Interest

The authors declare no conflicts of interest.

## Data Availability

The authors have nothing to report.
